# Harnessing serum VOCs and machine learning for the early detection of MAFLD

**DOI:** 10.3389/fendo.2025.1691853

**Published:** 2025-11-18

**Authors:** Xin Li, Xiaoyue Zhao, Ruonan Zhang, Xuewei Zhuang

**Affiliations:** 1 Shandong University of Traditional Chinese Medicine, Jinan, China; 2 Shandong University Affiliated Shandong Provincial Third Hospital Department of Clinical Laboratory, Jinan, China

**Keywords:** metabolic dysfunction-associated fatty liver disease, volatile organic compounds, gas chromatography–ion mobility spectrometry, machine learning, biomarker, early diagnosis

## Abstract

**Introduction:**

Metabolic dysfunction-associated fatty liver disease (MAFLD) is a complex metabolic disorder and one of the leading causes of chronic liver disease worldwide. Current diagnostic tools, such as ultrasound, lack sufficient sensitivity for detecting early-stage disease, emphasizing the urgent need for novel and non-invasive diagnostic strategies. Metabolomics, particularly the profiling of volatile organic compounds (VOCs) in biofluids, has emerged as a promising approach for biomarker discovery in metabolic diseases.

**Methods:**

In this preliminary single-center study, serum samples were collected from 199 participants, including 110 MAFLD patients and 89 healthy controls. Volatile organic compounds were analyzed using gas chromatography–ion mobility spectrometry (GC-IMS). Machine learning algorithms, including random forest, were applied to construct diagnostic models and identify key discriminatory metabolites. Clinical and biochemical parameters such as age, body mass index, liver function, and lipid profiles were also compared between groups.

**Results:**

A total of 79 serum VOCs were detected, among which 54 showed significant differences between MAFLD patients and controls (29 identified and 25 unidentified). The random forest model exhibited the best diagnostic performance, achieving a test AUC of 0.941, with 86.7% sensitivity and 88.5% specificity. Seven key VOCs were identified as important contributors to the model, including two upregulated compounds (2-Butoxyethanol and Cyclopentanone-D) and five downregulated compounds ((E)-3-hexenoic acid, 2-Ethylbutanal, 2-Propyl acetate, Benzaldehyde-M, and Furaneol). Notably, 2-pentylfuran displayed significant variation across different pathological grades of MAFLD, suggesting potential as a stage-specific biomarker.

**Discussion:**

This study demonstrates that serum VOC profiling using GC-IMS combined with machine learning can effectively distinguish MAFLD patients from healthy individuals. The identified VOC signatures, particularly 2-pentylfuran, may serve as non-invasive biomarkers for MAFLD diagnosis and staging. However, due to the limited sample size and single-center design, these findings require validation in larger, multi-center, and longitudinal studies to confirm their clinical applicability, especially for early disease detection.

## 1 Introduction

Metabolic dysfunction-associated fatty liver disease (MAFLD) is a complex metabolic disorder arising from an intricate interplay of genetic susceptibility, metabolic disturbances, and environmental factors. An international consensus group recommended in 2020 that MAFLD should replace the historically used term “non-alcoholic fatty liver disease” (NAFLD) (1–3). It is a leading cause of chronic liver disease worldwide (4, 5), affecting approximately 25% of the global population. It is a common reason for abnormal liver function tests and can account for up to 90% of cases of asymptomatic elevation in alanine aminotransferase (ALT) and aspartate aminotransferase (AST) levels once other causes of liver disease are excluded (6). Substantial research shows that MAFLD can progress to severe outcomes, including end-stage liver disease and hepatocellular carcinoma (HCC) (7). Liver biopsy remains the gold standard for diagnosis, but its invasiveness and associated clinical risks limit routine use. Consequently, diagnosis typically relies on a combination of medical history, laboratory tests, and imaging. Ultrasound can reliably detect moderate-to-severe steatosis and is currently the preferred imaging technique for screening in clinical and population-based settings (8). On ultrasound, the diffuse fatty infiltration of MAFLD often produces a hyperechoic texture, or “bright liver.” However, MAFLD is often asymptomatic in its early stages. When it comes to early detection, conventional methods have limited utility, and there is an urgent need for novel, non-invasive diagnostic methods.

Metabolomics, i.e., the systematic identification and quantification of small-molecule metabolites (<1500 Da) in a biological sample, offers a promising approach. The metabolome, or the complete set of metabolites in a biological system, provides a functional snapshot of the physiological state (9). Volatile organic compounds (VOCs), a subset of the metabolome, have emerged as powerful biomarkers. They can serve as non-invasive indicators of an individual’s metabolic status, and over the past decades, the VOC profiles of diverse biological fluids, including urine, breath, sweat, and feces, have been linked to various physiological and pathological states (10–13). For example, diagnostic models have been developed to identify cholangiocarcinoma using bile VOCs (14) and HCC using urinary VOCs (15). In addition, combining data from different sample types, such as blood and urine, has been shown to enhance the discriminative power and accuracy of diagnostic models (16).

Some researchers explored VOC signatures for NAFLD using breath and urine, each with distinct cohorts, platforms, and key findings. Shen et al. (17) applied 10 machine learning algorithms to 341 exhaled VOCs in a large cohort (n = 1,501). They showed that adding breath VOCs to demographic and biochemical predictors significantly improved NAFLD classification, identified specific breath compounds (e.g., 2-propanol, acetone) as influential features, and discussed environmental and exposure confounders and the need for external validation. Cozzolino et al. (18) profiled urinary VOCs in cohorts with NAFLD, type 2 diabetes, and their coexistence. They reported a set of urinary volatiles that discriminated disease groups and highlighting links to altered amino acid and lipid metabolism and noted that urine VOCs may reflect systemic metabolic changes rather than liver-specific processes. Skarysz et al. (19) developed convolutional neural networks to detect VOCs directly from raw GC−MS breath data, bypassing expert-led deconvolution and demonstrating automated detection of more compounds with high specificity and far shorter processing time. The CNN pipeline, evaluated on 120 clinical samples targeting 30 VOCs, outperformed conventional expert analysis and produced robust, scalable VOC lists suitable for rapid breath−based biomarker discovery. While these methods have demonstrated impressive success, breathomics can be influenced by environmental exposures, and urinary analyses may reflect systemic rather than liver-specific metabolic changes. Indeed, Masoodi et al. (20) reviewed metabolomics and lipidomics in NAFLD and emphasized the need for multi-matrix validation. They warned that the variability in sampling, analytic workflows, and identification confidence tends to limit cross-study comparability, and argued for orthogonal confirmation of VOC candidates by MS-based techniques and standardization of collection/analysis procedures. Therefore, in this study, we turned to serum VOCs instead, which may more directly reflect hepatic metabolic processes.

Headspace solid-phase microextraction (HS-SPME) coupled with gas chromatography–mass spectrometry (GC-MS) has proven to be a simple, rapid, and effective technique for analyzing VOCs in biofluids. This approach pairs chromatographic separation with mass spectral identification and extensive libraries for definitive compound assignment, but for large clinical screening, it is limited by long run times, intensive sample preparation, reliance on operator expertise, moderate throughput, high capital and per−sample costs, and occasional poor sensitivity or ambiguous identification for trace, highly polar, or coeluting breath VOCs. In contrast, gas chromatography–ion mobility spectrometry (GC−IMS) addresses these limitations by adding an orthogonal ion−mobility (drift−time) separation that improves resolution of isobaric/coeluting species, thus offering enhanced sensitivity for low−abundance metabolites, faster analyses with simpler headspace workflows, a smaller bench−top footprint, and lower operational burden. It can be readily integrated into clinical laboratory workflows and is particularly suitable for a large cohort study focusing on clinically deployable, high−throughput screening (21). Hence, in this work, we used GC−IMS to compare serum VOC profiles between MAFLD patients and healthy controls, to characterize disease-related metabolic changes and construct a novel, serum-based diagnostic model for MAFLD.

## 2 Materials and methods

### 2.1 Study design and participants

**Figure 1:**
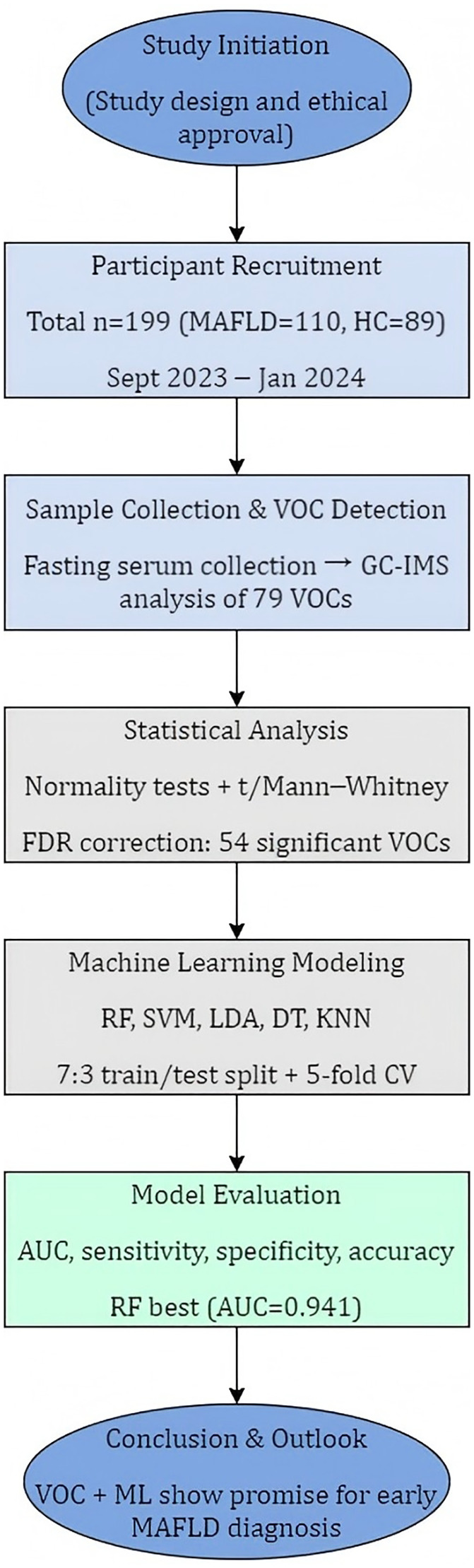
Research design schematic.

**Figure 1** illustrates the overall study design. A cohort was recruited between September 2023 and January 2024 from the Department of Physical Examination at Shandong Provincial Third Hospital. To minimize selection bias in this non-randomized study, we enrolled consecutive, eligible patients presenting to the hepatology clinic. Healthy controls were recruited from routine health check-up programs and were required to have normal liver imaging, normal liver enzyme levels, and no history of chronic liver or metabolic disease.

As our study’s inclusion criteria aligned with the 2020 MAFLD definition, we use the term MAFLD throughout this manuscript to most accurately describe our patient cohort. MAFLD grade (mild, moderate, or severe) was determined using standard ultrasound criteria, including hepatic echogenicity, clarity of intrahepatic vasculature, diaphragm visualization, and posterior beam attenuation.

#### 2.1.1 Inclusion criteria for the MAFLD group

1. Age between 18 and 75 years.
2. Evidence of hepatic steatosis on imaging studies or liver biopsy.
3. Presence of at least one of the following conditions:
  - Overweight or obesity (Body Mass Index [BMI] ≥23 kg/m^2^ for Asians).
  - Diagnosed type 2 diabetes mellitus.
  - Evidence of metabolic dysfunction, defined as the presence of at least two of the following seven traits:
    - Waist circumference: ≥90 cm (male) or ≥80 cm (female) for Asians.
    - Blood pressure: ≥130/85 mmHg, or use of antihypertensive medication.
    - Triglycerides: ≥1.70 mmol/L, or use of lipid-lowering medication.
    - High-density lipoprotein cholesterol (HDL-C): <1.0 mmol/L (male) or <1.3 mmol/L (female), or use of lipid-modifying medication.
    - Prediabetes: Fasting glucose 5.6–6.9 mmol/L, 2-hour postprandial blood glucose 7.8–11.0 mmol/L, or glycated hemoglobin (HbA1c) 5.7%–6.4%.
    - Homeostasis model assessment of insulin resistance (HOMA-IR) index: ≥2.5.
    - High-sensitivity C-reactive protein (hs-CRP) level: ≥2 mg/L.

#### 2.1.2 Inclusion criteria for the healthy control group

1. Age ≥18 years and provided voluntary written informed consent.
2. No history of major cardiac, hepatic, renal, neurological, or infectious disease.
3. BMI within the normal range (e.g., 18.5–22.9 kg/m^2^).
4. No heavy alcohol consumption, smoking, or any substance abuse.
5. No use of medications known to affect metabolic outcomes within the past month.
6. Not pregnant or breastfeeding.

### 2.2 Sample preparation

Peripheral blood samples were collected from all participants in the morning after an overnight fast. Samples were drawn into serum separator tubes containing a clot activator and centrifuged at 3000*g* for 10 min at 4°C within two hours of collection. The resulting serum was transferred to a new tube and centrifuged again at 12,000*g* for 10 min at 4°C to remove residual cellular debris. The final serum (supernatant) was aliquoted into cryotubes, flash-frozen, and stored at −80°C until analysis. Each sample was thawed no more than twice before analysis. To minimize exogenous contamination and avoid systematic bias, blood collection and processing were performed in a standardized environment using identical consumables for all samples.

### 2.3 GC-IMS analysis of serum VOCs

For each analysis, a 200 μL serum aliquot was placed in a 20 mL headspace vial, sealed, and incubated at 80°C for 10 min. Following incubation, 1 mL of the headspace gas was automatically injected into a GC-IMS system (FlavourSpec, G.A.S., Dortmund, Germany). The system used nitrogen as both the carrier and drift gas. The carrier gas flow was programmed as follows: 2 mL/min for 2 min, ramped linearly to 100 mL/min over 8 min, and then held at 150 mL/min for an additional 5 min. The IMS drift gas flow was constant at 150 mL/min. The drift tube, GC column, and inlet temperatures were maintained at 45, 60, and 60°C, respectively.

VOC signals were characterized by their retention index (RI), drift time (DT), and peak height (PH). Compounds were identified by comparing their spectral features against the National Institute of Standards and Technology (NIST) and integrated GC-IMS library databases, and external reference standards were used when appropriate. Peaks that could not be matched were numbered and reported as unidentified VOCs. Peak height was used for relative quantification across all samples.

### 2.4 Statistical and bioinformatic analysis

Descriptive statistics were calculated for all baseline characteristics. Continuous variables were presented as mean ± standard deviation (SD) or median [P25, P75] based on their distribution, assessed using the Shapiro–Wilk test. Categorical variables were presented as counts and percentages. For comparisons of individual VOC concentrations between the MAFLD and control groups, we used the independent samples t-test for normally distributed data and the Mann–Whitney U test for non-normally distributed data. All tests were two-sided, and statistical significance was verified when p < 0.05.

To identify factors associated with MAFLD, we first performed univariate logistic regression. Variables with a p-value < 0.05 were then included in a multivariate binary logistic regression model to identify independent factors associated with MAFLD. For VOC feature selection, a multi-step filtering process was applied. First, we corrected for multiple comparisons using the Benjamini–Hochberg false discovery rate (FDR) method, with a significance threshold of q < 0.05. To further ensure robustness, we only retained features that also exhibited an absolute fold change of at least 1.5 between the MAFLD and control groups and were detected in at least 20% of samples in either group. VOCs that met all these criteria were selected as the final feature set for machine learning.

We developed five machine learning models to classify MAFLD status based on the significant VOCs: random forest (RF), support vector machine (SVM), linear discriminant analysis (LDA), decision tree (DT), and k-nearest neighbors (KNN). The full dataset was randomly partitioned into a training set (70%) and a test set (30%) using stratified sampling to maintain the case-to-control ratio. Model hyperparameters were optimized using five-fold cross-validation within the training set. The final, optimized models were then evaluated on the independent test set. Diagnostic performance was evaluated using receiver operating characteristic (ROC) analysis, and performance was assessed using the area under the curve (AUC). To evaluate model robustness, we conducted a sensitivity analysis by excluding participants with ages in the lowest and highest 5% of the distribution, re-training the models, and comparing the performance metrics with those from the full dataset. All statistical analyses were performed using SPSS 22 (IBM, Armonk, NY, USA) and R (version 4.3.3), and the caret and pROC packages were utilized for machine learning tasks. Default parameters were applied unless specified otherwise.

## 3 Results

### 3.1 Baseline clinical characteristics of the study cohort

The study cohort included 199 participants: 110 patients with MAFLD and 89 healthy controls (HC). While the groups were balanced for sex, height, and several laboratory parameters (total bilirubin, creatinine, blood urea nitrogen), the MAFLD group was significantly older and had a higher weight and BMI (p < 0.001 for all). As detailed in **Table 1**, the MAFLD patients also exhibited significant alterations in liver enzymes (ALT, AST, GGT) and lipid profiles (TC, LDL, TG) compared to the controls.

**Table 1:** Patient characteristics.

Characteristics	Healthy Control (n = 89)	MAFLD (n = 110)	p-value
Sex			0.317
Male	36 (40.45%)	37 (36.64%)	
Female	53 (59.55%)	73 (66.36%)	
Age (years)	37.0 [28.5, 49.5]	49.0 [37.0, 60.0]	<0.001
Height (cm)	166.30 [162.40, 173.50]	165.45 [159.68, 172.90]	0.270
Weight (kg)	63.40 [56.60, 72.45]	73.95 [66.10, 81.85]	<0.001
BMI (kg/m^2^)	22.90 [21.15, 24.85]	26.45 [24.40, 29.33]	<0.001
Lab test results			
ALT (U/L)	15.30 [11.75, 20.35]	20.75 [15.20, 31.28]	<0.001
AST (U/L)	18.70 [16.55, 21.85]	20.55 [17.50, 26.55]	0.002
GGT (U/L)	14.50 [11.30, 21.15]	22.10 [17.98, 31.90]	<0.001
TBIL (µmol/L)	12.60 [9.80, 17.55]	13.20 [10.08, 16.03]	0.681
TC (mmol/L)	4.54 [3.95, 4.88]	5.04 [4.44, 5.90]	<0.001
LDL (mmol/L)	2.73 [2.43, 3.09]	3.31 [2.83, 3.79]	<0.001
TG (mmol/L)	0.90 [0.68, 1.28]	1.67 [1.19, 2.41]	<0.001
GLU (mmol/L)	5.20 [4.90, 5.40]	5.45 [5.20, 6.00]	<0.001
Scr (µmol/L)	57.50 [52.20, 65.90]	57.00 [49.78, 66.30]	0.722
UA (µmol/L)	303.10 [274.10, 338.30]	364.8 [317.23, 431.65]	<0.001
BUN (mmol/L)	4.50 [3.85, 5.60]	4.75 [4.20, 5.50]	0.303

^§^Data are expressed as count (ratio) or median [P25, P75]. ALT, alanine aminotransferase; AST, aspartate aminotransferase; GGT, gamma-glutamyl transferase; TBIL, total bilirubin; TC, total cholesterol; LDL, low-density lipoprotein; TG, triglycerides; GLU, glucose; Scr, serum creatinine; UA, uric acid; BUN, blood urea nitrogen.

### 3.2 Serum VOC profiles distinguish MAFLD patients from healthy controls

**Figure 2:**
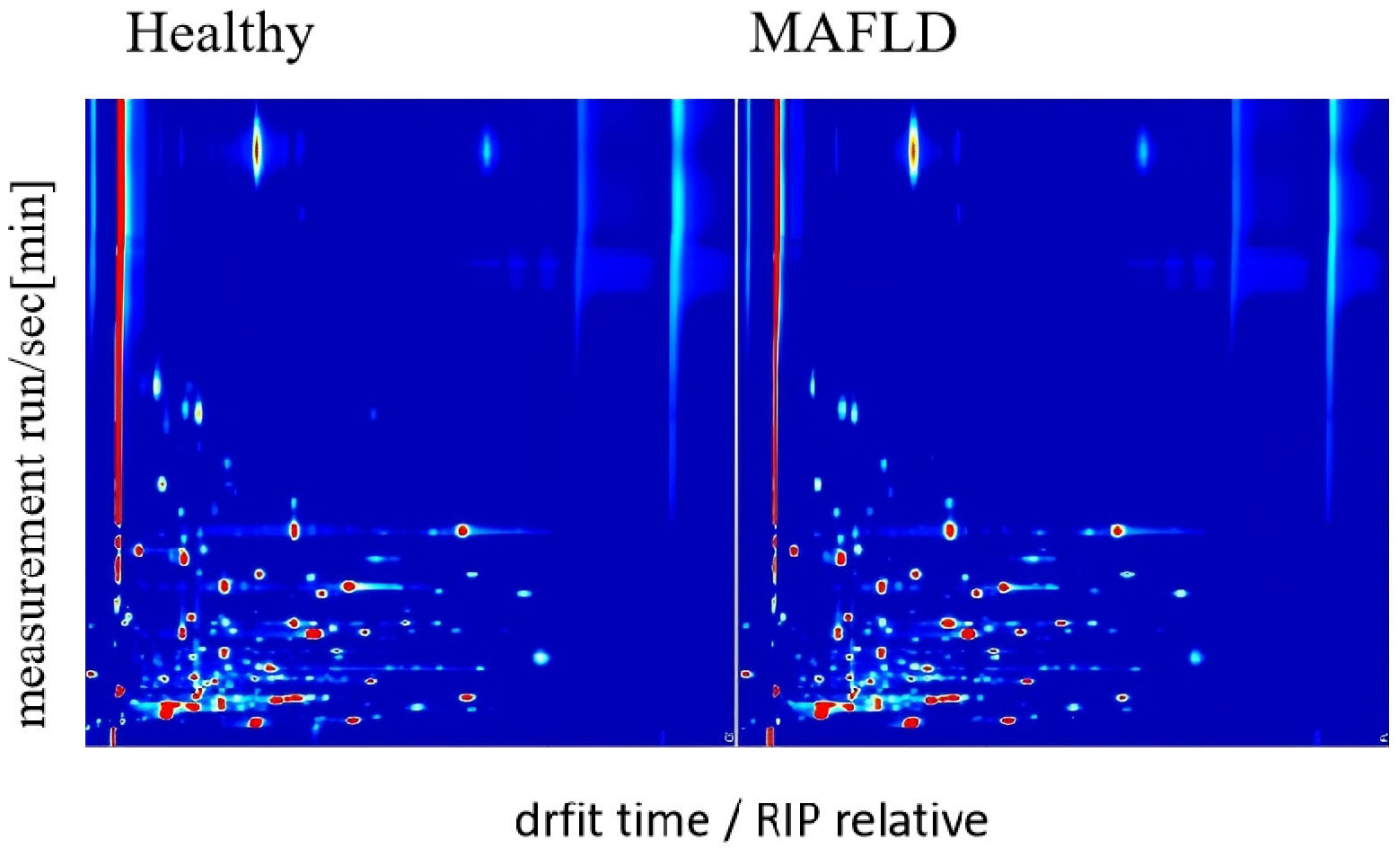
GC-IMS maps of serum VOCs in MAFLD patients (n = 110) and healthy controls (n = 89). The X-axis denotes retention index (a.u.), and the Y-axis denotes drift time (ms). Peak heights were log10-transformed, medians calculated per VOC across samples, then min–max normalized to [0, 1] for plotting. Color scale indicates normalized peak height (a.u.), with darker colors representing higher abundance.

The serum samples from all 199 participants were analyzed by GC-IMS to generate VOC profiles. Visual inspection of the resulting topographic plots revealed distinct differences in the VOC signatures between the MAFLD and HC groups (**Figure 2**). A total of 79 distinct VOC signals were quantified across all samples (**Supplementary Table S1**). Of these, 57 showed significant uncorrected differences between groups. After applying Benjamini–Hochberg (FDR) correction, 54 VOCs remained statistically significant (q < 0.05) and were selected as features for developing machine learning models (**Supplementary Table S2**). Some peaks were unidentified due to the current limitations of GC-IMS reference libraries, but they demonstrated consistent group differences and were thus retained for model construction.

### 3.3 Machine learning models achieve high diagnostic accuracy

**Figure 3:**
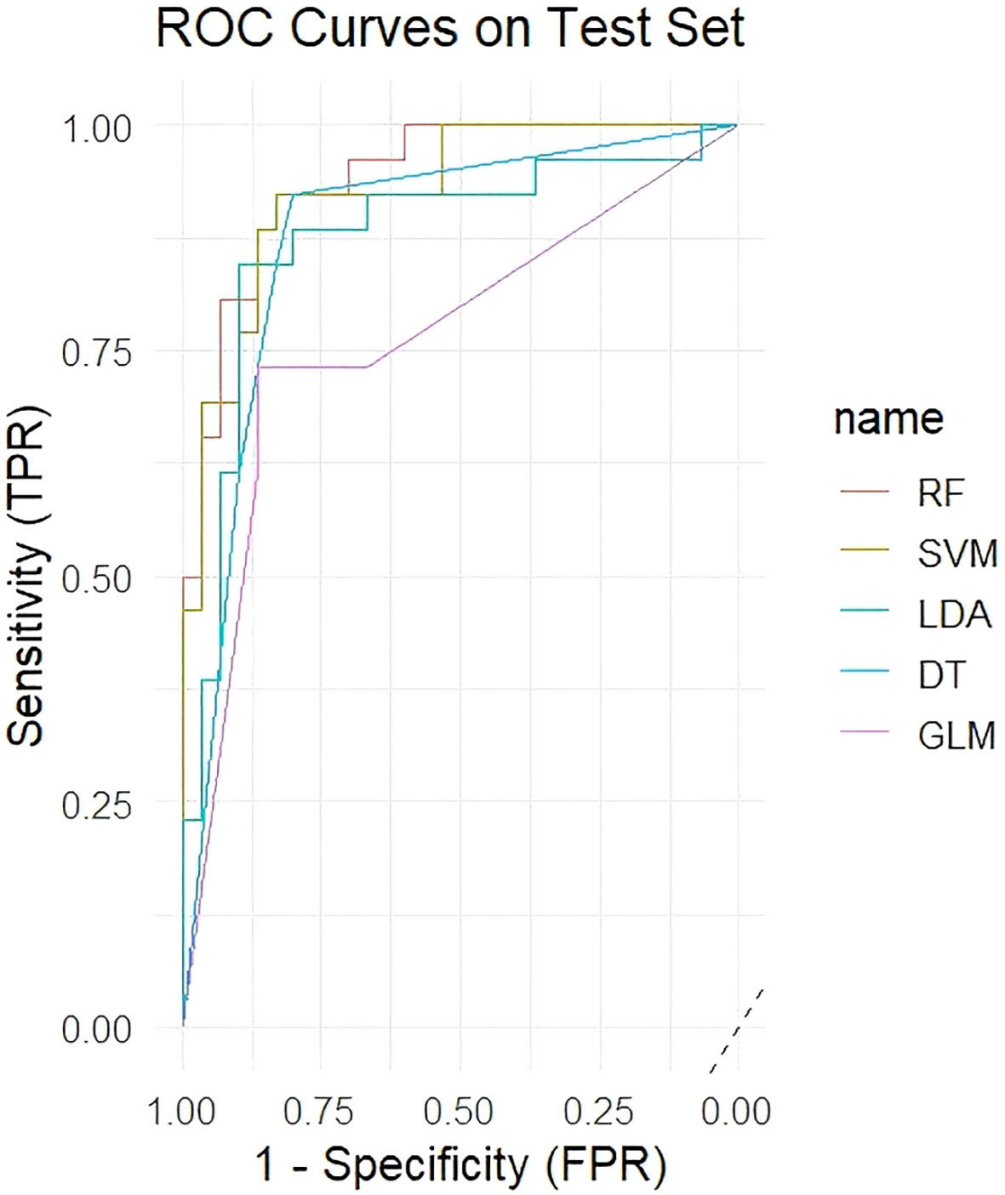
ROC curves of the five models on the test set.

**Figure 4:**
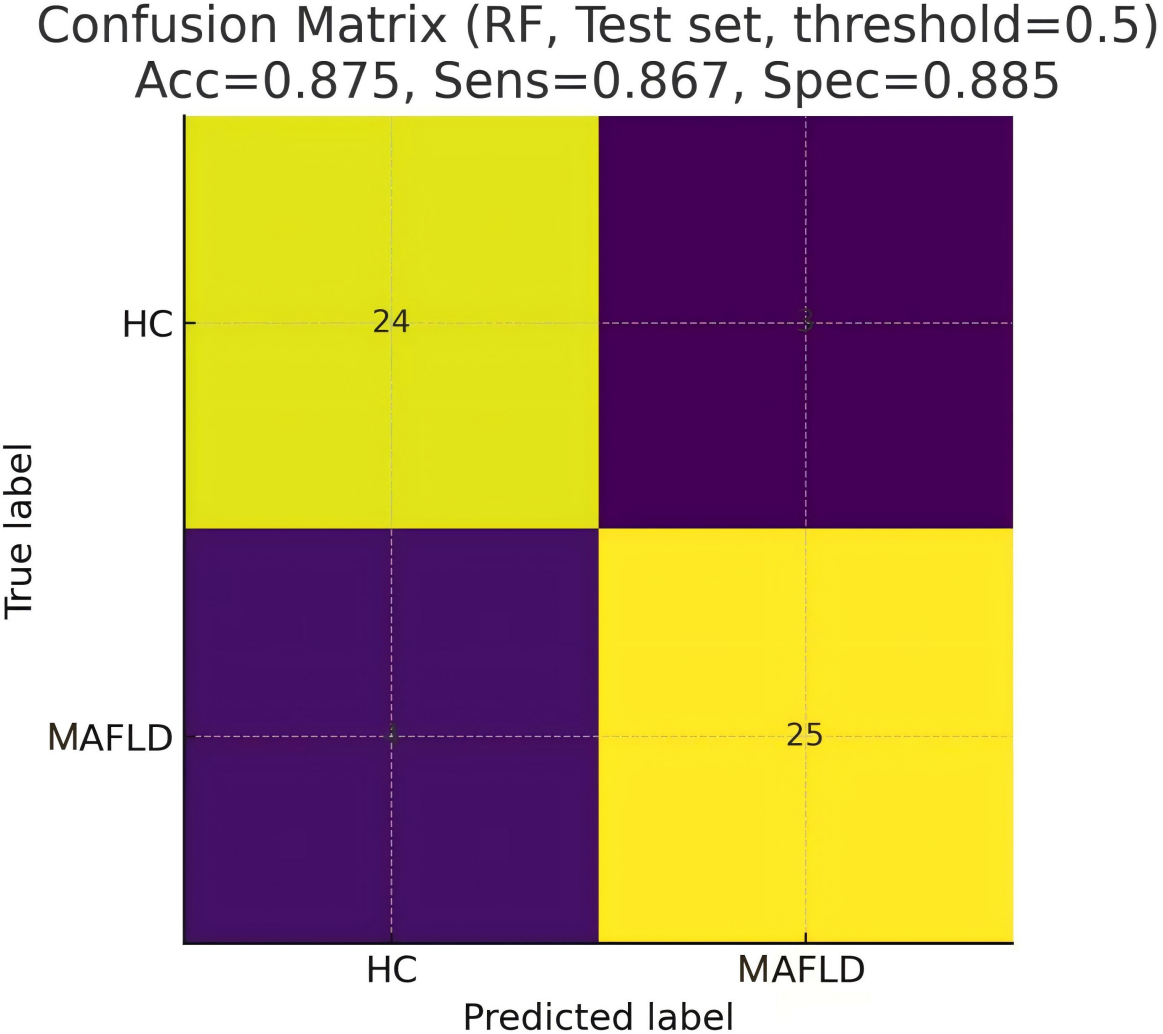
Confusion matrix of the RF model on the test set.

Five machine learning models were trained using the 54 significant VOC features, which included 33 identified and 21 identified compounds, and the diagnostic performance of the models was evaluated on an independent test set. **Table 2** shows that the RF model demonstrated the best performance, achieving an AUC of 0.941 on the test set, with an accuracy of 87.5%, sensitivity of 86.7%, and specificity of 88.5%. The SVM model also showed excellent performance (test set AUC = 0.927), whereas the LDA, DT, and GLM models were less effective. **Figure 3** shows the ROC curves of all the models on the test set. **Figure 4** shows the confusion matrix of the RF model on the test set.

**Table 2:** Diagnostic performance of machine learning models.

Model^§^	RF	SVM	LDA	DT	GLM
CV_AUC	0.936	0.896	0.83	0.848	0.692
Test_AUC	0.941	0.927	0.876	0.878	0.764
Acc_0.5	0.875	0.875	0.857	0.839	0.804
Sens_0.5	0.867	0.867	0.867	0.833	0.867
Spec_0.5	0.885	0.885	0.846	0.846	0.731
Thr_Youden	0.566	0.598	0.347	0.850	0.496
Acc_Youden	0.875	0.875	0.875	0.857	0.804
Sens_Youden	0.833	0.833	0.900	0.800	–
Spec_Youden	0.923	0.923	0.846	0.923	–

^§^RF, random forest; SVM, support vector machine; LDA, linear discriminant analysis; DT, decision tree; KNN. k-nearest neighbors. Metrics include area under the receiver operating characteristic curve (AUC), accuracy (Acc), sensitivity (Sens), and specificity (Spec) at default (0.5) and Youden’s index thresholds (Thr). All results reflect the diagnostic performance of binary classification between MAFLD and healthy control groups. The model performance was evaluated by cross-validation (CV) and using independent test sets.

**Figure 5:**
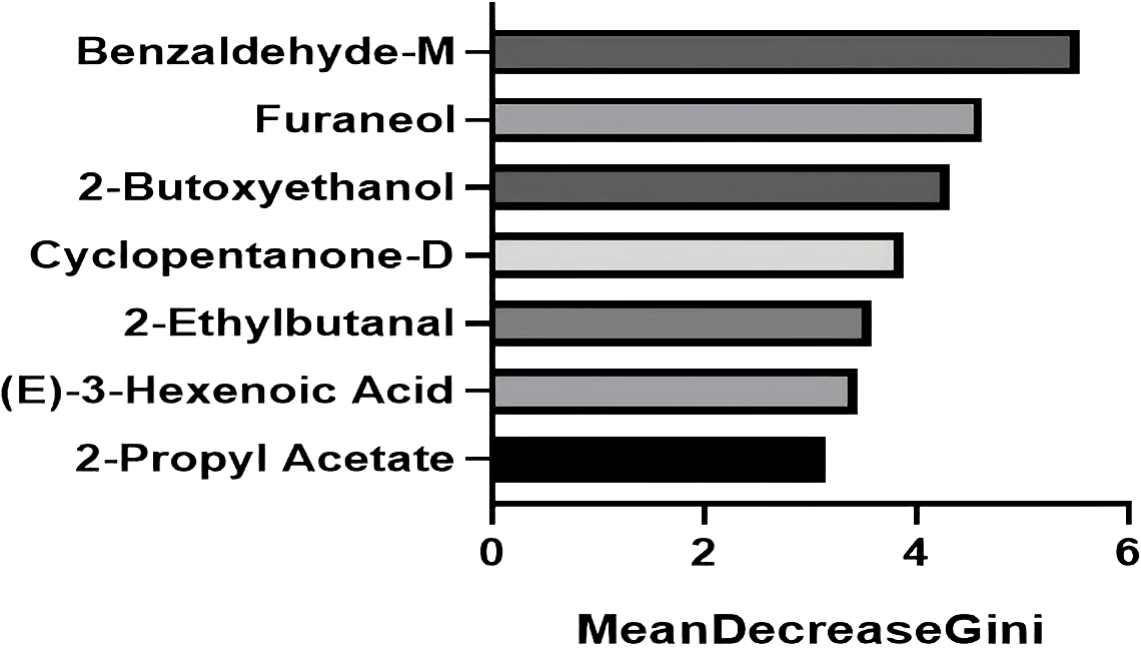
The top seven VOCs contributing most significantly to the predictive power of the random forest model. The bars represent the Gini coefficient calculated from the peak height of the respective VOC across all 199 patients. The seven selected VOCs all satisfy Gini coefficient ≥ 3.

**Figure 6:**
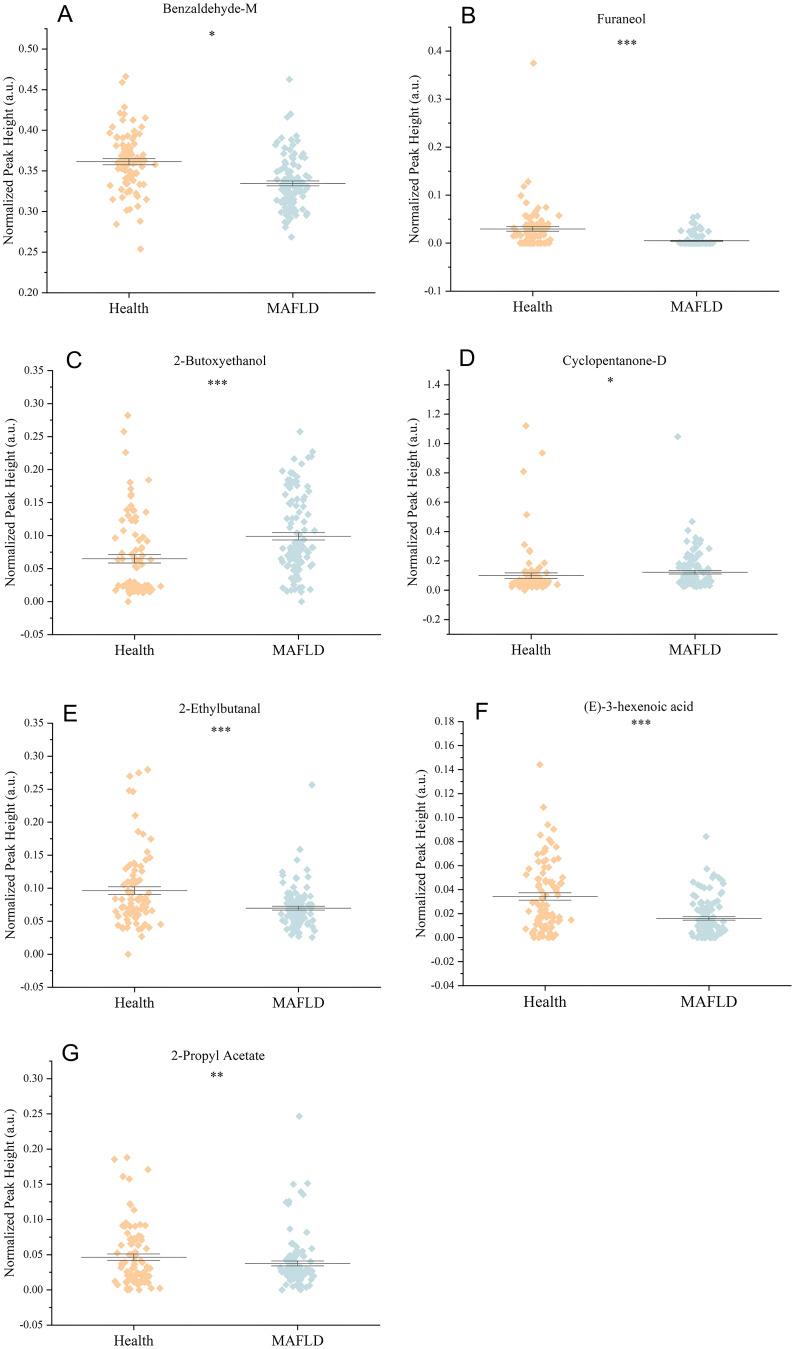
Comparison of the relative abundance (normalized peak height) of seven significantly altered VOCs between the Health and MAFLD groups. **(A)** Benzaldehyde-M, **(B)** Furaneol, **(C)** 2-Butoxyethanol, **(D)** Cyclopentanone-D, **(E)** 2-Ethylbutanal, **(F) (E)**-3-Hexenoic acid, and **(G)** 2-Propyl acetate. Data are shown as mean ± SEM. *p < 0.05, **p < 0.01, ***p < 0.001.

To identify the most influential biomarkers among the 54 significant VOCs, we calculated, for each VOC, the Gini coefficient of its peak−height distribution across all 199 subjects and then ranked features by their importance in the top-performing RF model. The analysis revealed seven key VOCs that contributed most significantly to the model’s predictive power (**Figure 5**). Among them, two compounds were upregulated (2-butoxyethanol, cyclopentanone-D) and five were downregulated ((*E*)-3-hexenoic acid, 2-ethylbutanal, 2-propyl acetate, benzaldehyde-M, furaneol) in the MAFLD group. **Figure 6** visualizes the distinct expression patterns of these top compounds. A sensitivity analysis that excluded age outliers confirmed the robustness of this VOC signature.

### 3.4 Association of VOCs with clinical parameters and disease severity

**Figure 7:**
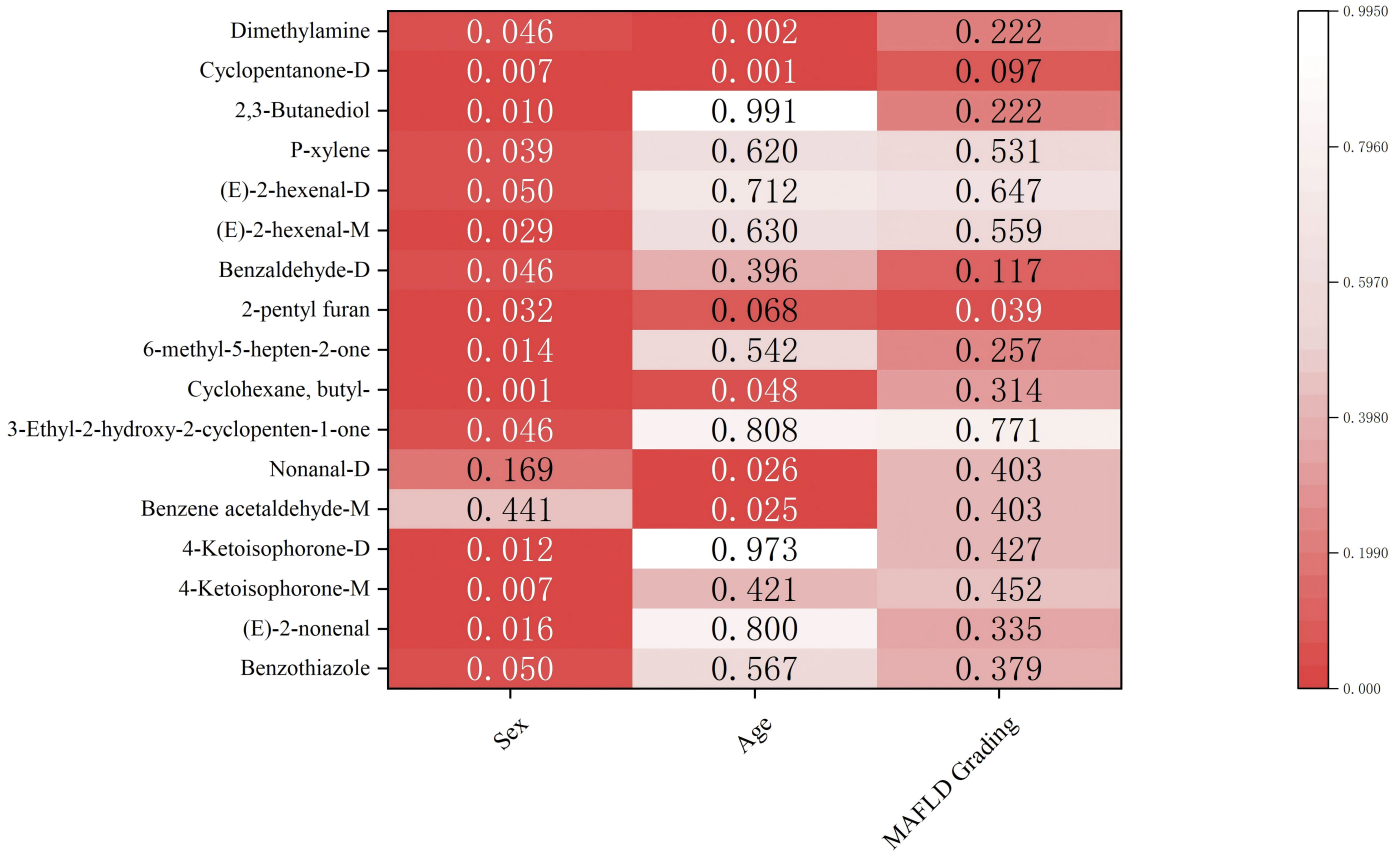
Associations between VOCs and pathological factors. Color intensity reflects −log10(p-value). Numbers colored in white denote statistically significant entries.

We next explored the association between the 33 identified significant VOCs and clinical parameters within the cohort. Numerous VOCs were significantly correlated with demographic factors such as sex and age (**Figure 7**). Notably, when analyzing the relationship with disease severity as determined by ultrasound, we found that the abundance of 2-pentyl furan varied significantly across mild, moderate, and severe stages of MAFLD (p < 0.05). This suggests that 2-pentyl furan may be a potential biomarker for monitoring MAFLD progression.

## 4 Discussion

VOC-based diagnostic model offers advantages such as convenient sampling, rapid detection, non-invasiveness, high patient acceptability, and the possibility of repeated sampling for dynamic monitoring (22, 23). This study demonstrates that a serum VOC-based signature, coupled with machine learning, can distinguish patients with MAFLD from healthy individuals with high accuracy. Current modalities of MAFLD diagnosis like ultrasound have limited sensitivity for early-stage disease, and our VOC-based model offers a promising, non-invasive alternative. The RF model, our top-performing algorithm, achieved an excellent AUC of 0.941, suggesting that serum VOCs reflect distinct metabolic perturbations in MAFLD. By identifying key VOCs linked to MAFLD, this work not only provides a potential diagnostic tool but also offers insights into the underlying pathophysiology of the disease.

A key strength of our study is the robustness of the VOC signal. Although the MAFLD and control groups had a significant age disparity, a sensitivity analysis excluding age extremes confirmed that the model’s performance was not driven by this demographic difference. Thus, the identified VOC signature must genuinely reflect disease-related metabolic changes. However, our findings should be interpreted with caution, since this work was a preliminary, single-center study with a limited sample size.

### 4.1 Comparison with existing literature and the novel contributions of this work

Our findings align with a growing body of research demonstrating the utility of VOCs as biomarkers in various diseases. For example, Hu et al. found a close relationship between urinary VOC metabolites and increased systemic inflammation, with smokers being more susceptible (24). This finding underscores the link between VOCs and inflammation—a key driver of MAFLD progression—and highlights the importance of accounting for lifestyle factors like smoking in metabolomic studies. Li et al. utilized GC-IMS to analyze VOC variations and explored the potential application of VOCs in the phenotypic detection of Carbapenem-Resistant *Klebsiella pneumoniae* (CRKP) strains (25). Previous studies also examined the use of VOCs as diagnostic biomarkers for esophageal cancer (26), pancreatic cancer (27), prostate cancer (28), esophageal adenocarcinoma (29), HCC (15), and colorectal cancer (30). A number of studies have investigated the diagnostic value of VOCs in liver disease (31), particularly in breath-based analyses. Breathomics approaches have been explored for HCC, cirrhosis, and MAFLD (32), with some technologies even advancing toward commercialization (e.g., VOC-based breath analyzers) (33, 34).

The combination of VOCs and machine learning has been deployed successfully in various scenarios. Fu et al. developed a machine learning model using urinary VOC metabolites and demographic data to predict cardiovascular disease risk (35). Their Random Forest model achieved a high predictive accuracy (AUC = 0.8143), which reflects our findings of the RF model and shows the power of combining metabolomic data with machine learning for risk stratification in complex metabolic diseases. While their model was effective, our serum-based approach in a hepatic disease context yielded an even higher AUC (0.941 vs. 0.814), highlighting the strong, tissue-proximal signal captured from serum. Thomas et al. (36) reported that machine learning analysis of exhaled VOC profiles can non-invasively detect cirrhosis and portal hypertension, thus offering a promising biomarker strategy for liver disease. Patnaik et al. (37) conducted a pilot study where they measured breath concentrations of isoprene, limonene, and dimethyl sulfide before and after exercise and used machine learning regression to predict liver−function scores. These studies all validate the overall approach.

Our work offers several unique contributions. First, unlike most prior MAFLD research that focused on exhaled breath or urine, we analyzed serum VOCs (17, 18). This sample matrix may more directly reflect endogenous hepatic metabolism and is less susceptible to confounding from environmental or dietary exposures (22). Second, our use of GC-IMS provided a rapid and highly sensitive platform suitable for clinical workflows (38). Third, by comparing multiple machine learning algorithms, we not only identified RF as the optimal model for this dataset but also demonstrated consistent discriminatory signals present in the VOC data, as even simpler models like GLM showed significant, albeit lower, performance (Test AUC = 0.764). This multi-model approach enhances the credibility of our findings compared to studies relying on a single algorithm. Finally, our model’s high performance (AUC = 0.941) suggests it has the potential to complement or even surpass traditional non-invasive markers like liver enzymes and lipid profiles, which may overlap between healthy and diseased states.

### 4.2 Biological and clinical interpretation of key VOCs

**Figure 8:**
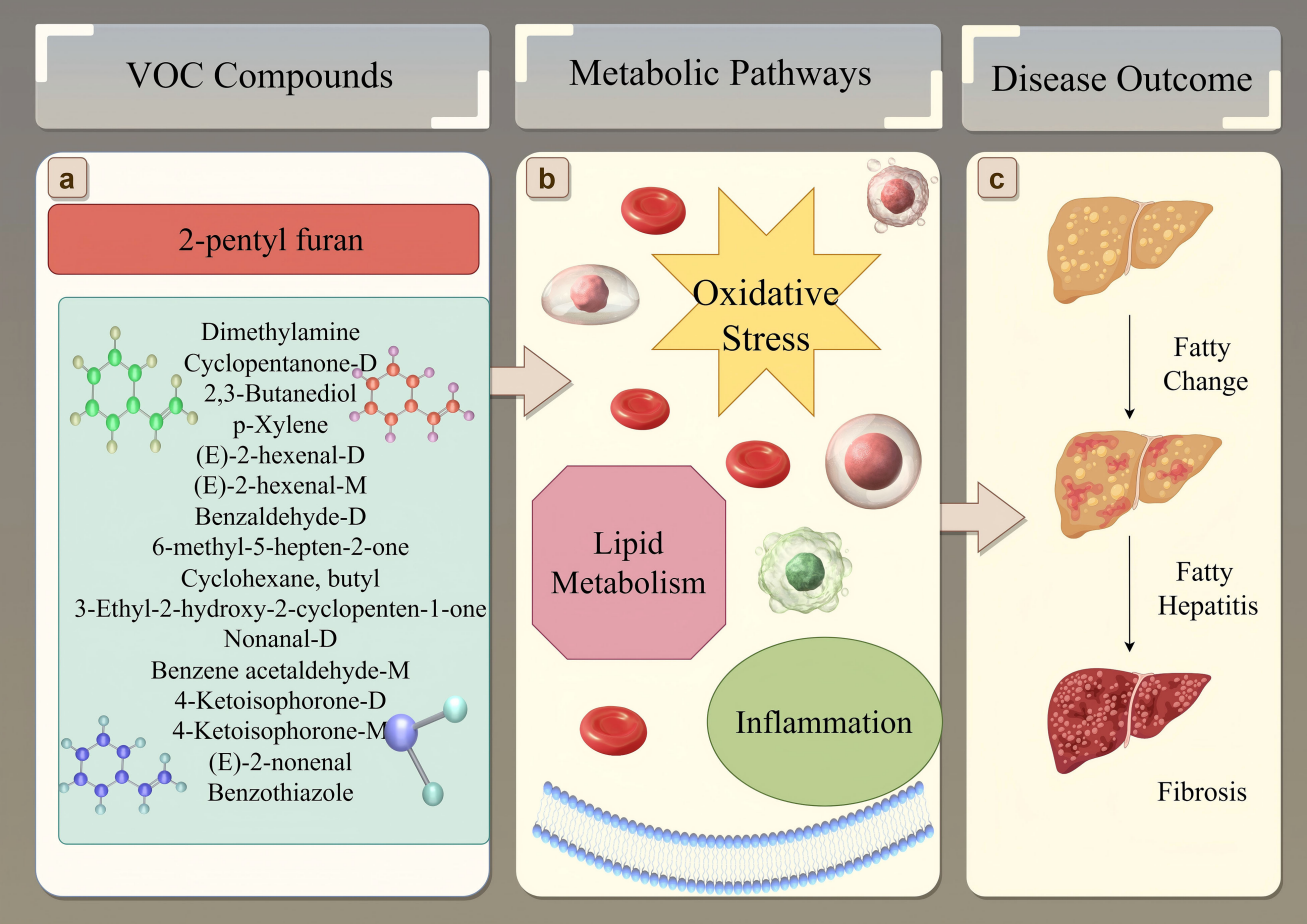
Schematic overview linking key VOCs to MAFLD pathogenesis. **(A)** Selected significant VOCs identified in this study, with 2-pentyl furan highlighted due to its association with disease severity. Chemical structures represent examples from the list. **(B)** These VOCs are putatively linked to core metabolic disturbances in MAFLD, including oxidative stress, altered lipid metabolism, and inflammation. **(C)** These metabolic changes contribute to the progression of liver damage from simple fatty change (steatosis) through fatty hepatitis (steatohepatitis) to fibrosis.

The VOC signature we identified reflects key metabolic hallmarks of MAFLD pathogenesis, including oxidative stress, disrupted lipid metabolism, and inflammation, as schematically illustrated in **Figure 8**. Our feature importance analysis identified seven VOCs with the highest discriminatory power, and their putative biological origins align with these processes. Our feature importance analysis identified seven VOCs with the highest discriminatory power. The VOC signature we identified dovetails with the hallmarks of MAFLD—oxidative stress, disrupted lipid oxidation, and altered xenobiotic processing. Of these seven key VOCs, 2-butoxyethanol is a CYP-metabolized solvent derivative, and its upregulation in MAFLD patients likely reflects enhanced hepatic detoxification activity in steatotic livers. The upregulation of cyclopentanone-D, a ketone byproduct of fatty-acid β-oxidation overload, reveals mitochondrial stress and lipid catabolism disruption. The downregulation of (*E*)-3-hexenoic acid and 2-ethylbutanal, both of which are α,β-unsaturated aldehydes formed during ω-6 lipid peroxidation, may indicate modified peroxidation flux or enhanced hepatic trapping/adduct formation. The downregulation of 2-propyl acetate and benzaldehyde-M, which are esters and aromatic aldehydes partly generated by the gut microbiome and amino acid metabolism, suggests dysbiosis and impaired hepatic clearance. Finally, the downregulation of furaneol, a furanone linked to Maillard/carbohydrate fermentation byproducts, may signal altered gut–liver crosstalk and glycation stress handling. Together, these patterns show that MAFLD involves not only lipid accumulation but also increased oxidative damage, impaired mitochondrial function, and changes in both host− and microbiome−derived volatile metabolites.

Interestingly, we observed significant associations between specific VOCs and demographic factors (**Figure 6**). Fifteen VOCs showed sex-specific differences, and six were correlated with age. For example, nonanal increased with age, which underscores cumulative lipid peroxidation and oxidative damage in MAFLD. Similarly, the sex-related difference in the ketone metabolite cyclopentanone-D may stem from hormonal regulation of mitochondrial fatty acid β-oxidation, thus suggesting that sex-specific metabolic pathways are involved in MAFLD. 2-Pentyl furan, highlighted in our analysis (**Figure 8A**) and known as an autoxidation product derived from linoleic acid (a polyunsaturated fatty acid), provides a direct link to lipid peroxidation and oxidative stress pathways implicated in MAFLD progression (**Figure 8B**). The most clinically relevant finding was its significant association with MAFLD severity graded by ultrasound. This correlation suggests that serum levels of 2-pentyl furan could potentially serve as a non-invasive biomarker for monitoring disease progression (**Figure 8C**) or stratifying patients based on severity, which would address a key unmet need in clinical practice. However, since we did not perform a parallel analysis of these demographic associations in the healthy control group, it remains unclear whether these effects are disease-specific or simply reflect baseline population variations.

### 4.3 Rationale for the machine learning approach

The superior performance of the RF model (Test AUC = 0.941) in our study can be attributed to its ability to handle high-dimensional data and model complex, non-linear relationships between variables without overfitting (39). By building an ensemble of decision trees, RF effectively reduces noise and automatically selects the most important predictive features (40). In contrast, DT is prone to overfitting and had a lower performance (Test AUC = 0.878), although it is simple to interpret (41). The SVM model also performed well (Test AUC = 0.927), as it is well-suited for high-dimensional data with small sample sizes, although it can be computationally intensive (42). The strong performance of these advanced algorithms, compared to the simpler GLM (Test AUC = 0.764), highlights the importance of capturing non-linear interactions among VOCs for accurate MAFLD diagnosis.

### 4.4 Limitations and future directions

This study has several limitations. First, the single-center design and relatively small sample size (n = 199) limit the generalizability of our findings. Second, the cross-sectional design prevents us from establishing a causal relationship between VOC changes and MAFLD progression. Third, our diagnosis and staging of MAFLD were based on ultrasound rather than liver biopsy, the gold standard. This prevents us from distinguishing between simple steatosis and the more aggressive non-alcoholic steatohepatitis (NASH). Fourth, several significant VOCs could not be identified due to the limited library coverage of GC-IMS, which hinders a complete biological interpretation. Finally, the demographic analysis was restricted to the MAFLD group, and we did not fully disentangle disease-specific effects from normal population variance.

Building on this work, future research should focus on several key areas.

#### 4.4.1 Clinical validation

Large-scale, multi-center, prospective studies are the highest priority. These studies should include diverse populations and use histopathology to confirm diagnoses, allowing for the validation of VOCs as biomarkers for both early detection and staging (i.e., steatosis vs. NASH vs. fibrosis). Longitudinal studies are also needed to track changes in serum VOCs over time in target populations.

#### 4.4.2 Mechanistic insight

Mechanistic studies are needed to understand the biological origins of the key VOCs identified. Combining metabolomics with other omics data (e.g., transcriptomics, proteomics) can help elucidate specific pathways (e.g., lipid metabolism, inflammation, gut–liver axis signaling) that are disrupted in MAFLD.

#### 4.4.3 Technological advancement

Continued optimization of VOC detection technologies and exploration of advanced machine learning algorithms, like deep learning, will further enhance model performance. Future studies can also benefit from integrating GC-IMS with GC-MS for more comprehensive compound annotation.

#### 4.4.4 Multimodal diagnostics

Research should explore how to integrate VOC profiling with existing diagnostic tools (ultrasound, blood tests, FibroScan) to create a more powerful, multi-modal strategy for MAFLD management, as such an approach should improve diagnostic accuracy and support personalized treatment decisions.

## 5 Conclusions

In summary, this study demonstrates that a serum VOC signature, when analyzed by machine learning, can accurately distinguish patients with MAFLD from healthy controls. Our model demonstrated high diagnostic performance and identified several promising biomarkers potentially linked to disease severity. However, the findings are constrained by the study’s single-center design and lack of histopathological validation. Further validation in large, prospective cohorts is required to confirm the clinical utility of this approach for the early detection and management of MAFLD.

## Data Availability

The original contributions presented in the study are included in the article/**Supplementary Material**. Further inquiries can be directed to the corresponding author.
