# Supplementary figures and images for: Harnessing serum VOCs and machine learning for the early detection of MAFLD
Source: Front Endocrinol (Lausanne). 2025 Nov 18;16:1691853. doi: 10.3389/fendo.2025.1691853 (PMC12668926; doi:10.3389/fendo.2025.1691853)

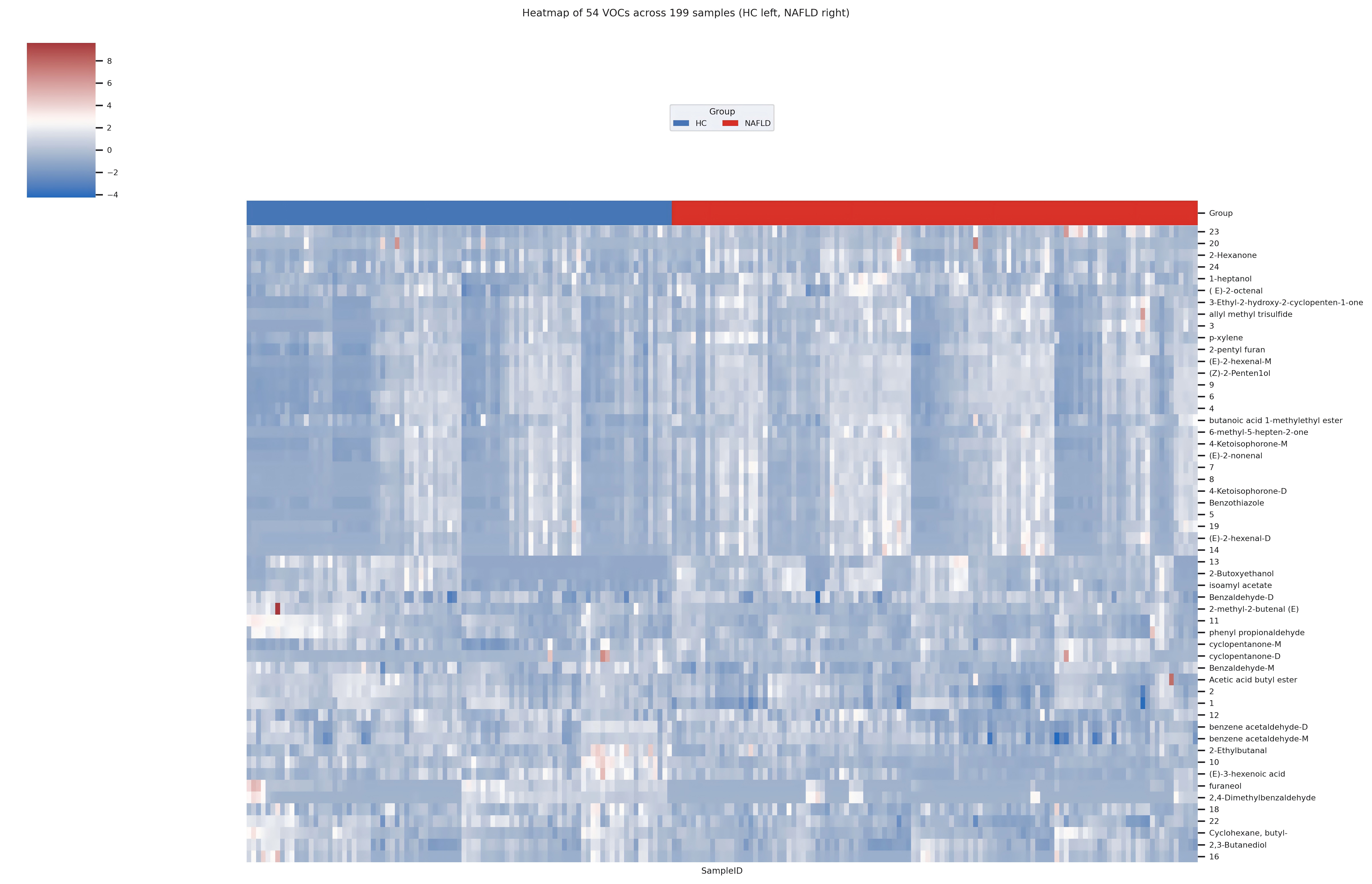

Supplement: Supplementary file 1 [file Image1.jpeg]

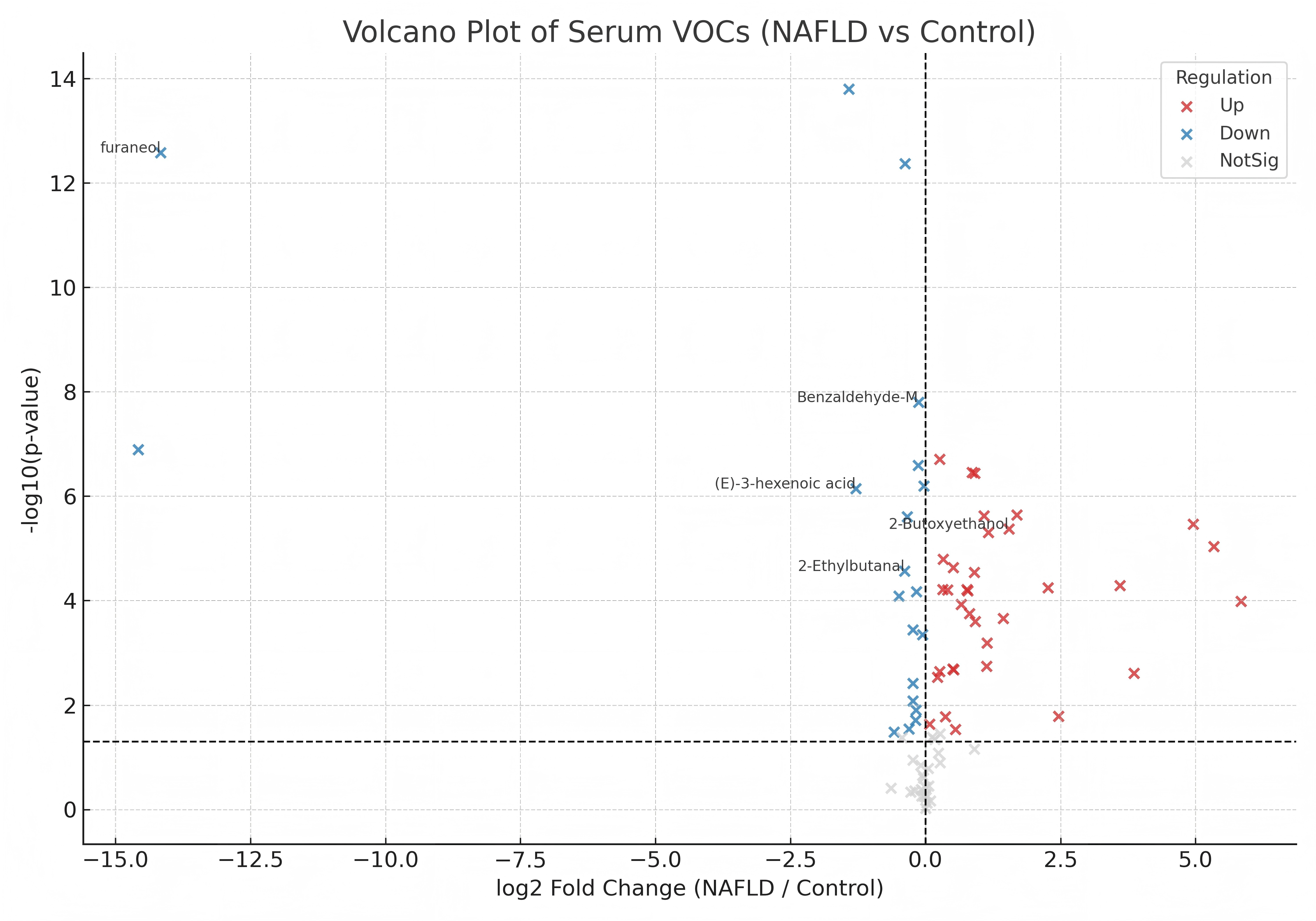

Supplement: Supplementary file 2 [file Image2.jpeg]
